# Evaluation of disc degeneration adjacent to AOspine A fractures: pre- and post-operative MRI analysis

**DOI:** 10.1051/sicotj/2020032

**Published:** 2020-08-28

**Authors:** Laura Marie-Hardy, Nicolas Barut, Hedi Sari Ali, Marc Khalifé, Hugues Pascal-Moussellard

**Affiliations:** 1 Orthopedic Surgery Department, Pitié-Salpêtrière Teaching Hospital 47 bd de l’Hôpital 75013 Paris France

**Keywords:** Spine, Fractures, Osteosynthesis, MRI, Discs

## Abstract

*Introduction*: The management of type A thoracolumbar fractures varies from conservative treatment to multiple level fusion. Indeed, although Magerl defined the type A fracture as a strictly bone injury, several authors suggested associated disc lesions or degeneration after trauma. However, the preservation of mobility of the adjacent discs should be a major issue. This study was conducted to analyze the presence of immediate post-traumatic disc injuries and to know if discs degenerate after receiving treatment. *Methods*: We retrospectively reviewed the files of 27 patients with an AOspine A fracture, corresponding to 34 fractures (64 discs) with pre and post-operative MRI (mean follow-up: 32.4 months). Based on Pfirrmann’s and Oner’s classifications of disc injuries, two observers analyzed independently the type of lesion in the discs adjacent to the fractured vertebra in immediate post-trauma and at the last follow-up. *Results*: The immediate post-traumatic analysis according to Pfirrmann’s classification found 97% of the cranial adjacent discs and 100% of the caudal discs classified Pfirrmann 3 or less. The analysis on the secondary MRI revealed that 78% of cranial adjacent discs and 88% of caudal adjacent discs still were classified Pfirrmann 3 or less. *Conclusions*: Since, the great majority of type A fractures does not cause immediate disc injuries, these fractures are, as described by Magerl, strictly bony injuries. The quality of the body reduction seems to prevent secondary degeneration. These results may encourage surgeons not to perform arthrodesis on type A fractures even for A3 and A4.

## Introduction

Thoracolumbar fracture incidence is increasing, constituting both a therapeutic and an economic challenge [1]. Magerl et al. reported the most used classification of these fractures, in three types (A, B, and C with sub-divisions), which has evolved into the AOspine classification [2, 3] Type A fractures are the result of a mechanism in compression, type B of flexion combined with distraction, and type C of rotation. It is commonly accepted that considering the presence of unstable and definitive disco-ligamentous injuries in types B and C, performing an arthrodesis is logical in these cases [4]. However, for type A fractures which are the most common, there is no therapeutic consensus, and treatment varies according to the authors, ranging from conservative treatment with a cast to a surgical procedure with a vertebroplasty, using an open or a percutaneous fixation, and sometimes a corporectomy and a fusion are performed [5, 6].

Type A fractures are mainly found in young patients and at the thoracolumbar junction and below. That is why preservation of discs and segmental mobility is so crucial. Determining the disc status appears to be an essential element to define the appropriate treatment for these fractures.

Historically, for Magerl, type A fracture was a strictly bony and stable injury [2]. Nonetheless, several studies have highlighted a loss of sagittal correction after fracture treatment, whether it was conservative or surgical [7–9]. This loss of correction was evaluated around 10% and was located in the disc for 75% and in the vertebral body for the remaining 25%. However, most of these later measurements were performed on plain radiographies which have been correlated to a lower accuracy than the measurements performed with CT-scans. This lack of accuracy may suggest false disc injuries. Looking at the literature, several studies focused on the MRI signal of discs adjacent to the fractured vertebra and their results are contrasted, notably due to their heterogenicity in terms of design (number of MRI, type of fractures analyzed…). First, in 1998, Oner et al. analyzed the MRI signal of discs adjacent to 75 fractures, and designed a classification with six different patterns of disc injuries [10]. They observed that these fractures were regularly associated with post-traumatic disc lesions.

However, it was a retrospective study based on MRI performed very lately after trauma. Moreover, among the 75 fractures that were analyzed, only 58 were type A, and 17 were types B and C. According to Pfirrmann classification, only Oner’s types 2 and 6 correspond to a degenerative change of the signal, the types 3, 4, and 5 correspond to morphologic changes that are not prejudging the functional quality [11]. However, in 2013, this same team published a study of 20 patients with type A3 fractures treated with short-segment pedicle screw instrumentation without fusion [12]. An immediate post-traumatic MRI and another one after implant removal at 12–18 months post-trauma were performed. They showed that discs were not injured initially and only 13% were progressing toward degeneration. Wang et al. conducted a similar study on 26 patients with type A3 fractures treated by percutaneous fixation and systematic implant removal between 9 and 12 months [13]. They concluded that 92% of the cranial adjacent discs degenerated (mean stage: 2.1–3.4) according to Pfirrmann classification [11]. But conversely, Alanay et al. in 2004 found morphologic changes, but without signal intensity change (Oner 1) on the MRI of 15 patients treated conservatively for AOspine A3–A4 lumbar fractures [14]. Finally, Loriaut et al. in 2015 analyzed 95 type A fractures treated conservatively or surgically and did not find significant loss of disc height or MRI signal modification of the adjacent discs, cranial or caudal [15].

Do the adjacent discs in type A fractures present injuries in immediate post-trauma and are they evolving to degeneration? Is the situation different for cranial and caudal adjacent discs? If some discs show a certain level of degeneration, what may be the explanation? Regarding these results, how should type A fractures be managed?

This study was therefore performed to investigate the presence of injuries in the adjacent discs in immediate post-trauma and to evaluate whether or not these discs evolve to degeneration after AOspine A fractures.

Our hypothesis was that AOspine A fractures were not associated to discs injuries in immediate post-trauma, and secondary disc degeneration occurred only in a low rate of patients with no need for secondary arthrodesis.

## Methods

The study design was retrospective on data collected prospectively. The inclusion criteria were AOspine A fractures treated in our orthopaedic department between 2007 and 2017 and having both preoperative CT-scan and MRI and a post-traumatic MRI at a minimum follow-up of 6 months. Type B or C fractures, above T5 or below L5, fractures on ankylosed spine, and osteoporotic fractures (resulting from a low-kinetic mechanism or confirmed with bone densitometry) were excluded from the analysis.

We collected the usual demographic data (age, sex) and comorbidities (in order to exclude fractures on pathologic spine), data on the fractures (number of fractured vertebrae, number of adjacent discs to the fractured vertebrae, type according to AOspine classification), and clinical data (Frankel status) for every patient [2, 3]. On the initial lateral radiographs, we measured the local kyphosis (LK) which is the angle between the superior and inferior endplates of the fractured vertebra and the regional kyphosis (RK) which was defined as the angle between the superior endplate of the superior uninvolved vertebra and the inferior endplate of the inferior uninvolved vertebra. We calculated the regional traumatic angle (RTA) which is equal to the regional kyphosis minus the physiological angulation (PA) expected at the fractured level (RTA = RK – PA) [16]. Physiological angulation values at the different levels have been previously expounded by Guigui et al. [17]. The same measurements were performed at the end of immobilization (conservative group of patients) or in postoperative and during follow-up (surgical group).

According to Oner’s and Pfirrmann’s classifications, two observers independently reviewed the signal intensity of the adjacent disc to the fractured vertebrae on sagittal T2 MRI images [10, 11]. For each disc was assigned a type of lesion according to the five patterns described by Pfirrmann [11] and the six patterns previously described by Oner et al. [10]. For the analysis, the values of the senior observer were taken into account. The same analysis was performed on the second MRI to assess stability or degeneration of the adjacent discs. Several parameters such as age, sex, local kyphosis, and regional traumatic angle were analyzed to determine whether they were predictive factors for initial or secondary disc injury. Pfirrmann’s classification was chosen as the main evaluation criterion due to its higher reproductivity [11, 18]. Disc degeneration according to Pfirrmann’s classification was analyzed in two groups: 1–3: mild-to moderate degeneration and 4–5: severe-to-end stage, in accordance with previous studies, but the detailed results are shown in this study [11, 12]. The results of the analysis according to Oner’s classification are shown in order to have an external control of our findings.

This analysis used the Fischer exact test for categorical variables and the Wilcoxon test for quantitative variables (not normally distributed). The significance level was set at *p* < 0.05. The software used for the analysis was RStudio (*AGPL v3, USA*).

## Results

Thirty-four fractures were analyzed in 27 patients with an Aospine A fracture for a total of 64 discs investigated. The cohort consisted of 16 men and 11 women (sex-ratio: 0.59) with a mean-age of 43.9 years. A total of 27 patients were studied, corresponding to 34 fractures. Twenty-two patients had a single fracture and six had two simultaneous fractures (Table 1). The mean-delay for the first MRI was 0.4 months (±0.7; [0.0; 2.0]) and it was 32.4 months (±34; [3; 117]) for the second MRI. Two patients were treated conservatively with casting for 6 weeks, 25 had surgery with short-segment pedicle screw fixation without fusion (9 percutaneous-fixation and 16 open-fixation). After assessing the fracture consolidation on a CT-scan, implants were removed within 9–12 months after surgery.

**Table 1:** General characteristics of the cohort.

	Characteristics of the cohort		
Age (years)	Mean	SD	Min–max
	43.9	15.3	[18; 79]
Sex	Female (%)	Male (%)	
	41	59	
Number of fractures (%)	One (%)	Two (%)	
	78	22	

**Figure 1:**
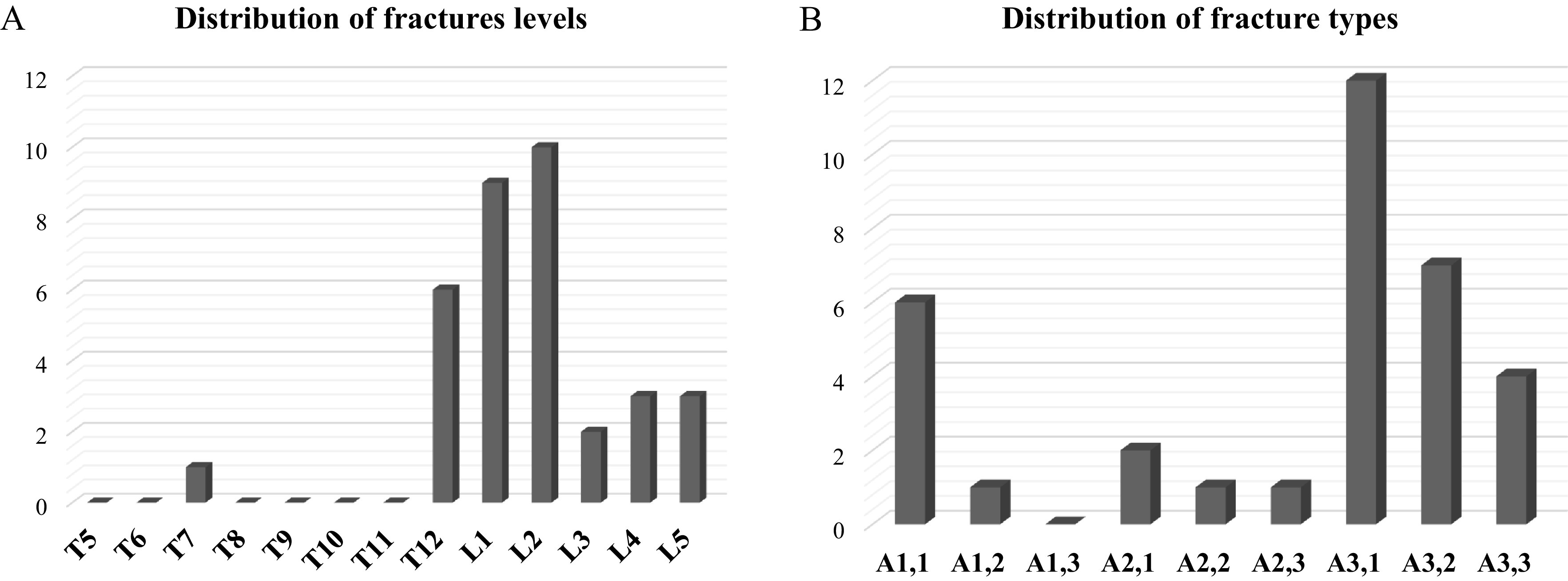
(A) Histogram showing the distribution of fracture levels. (B) Histogram showing the distribution of fracture types according to the AOspine classification.

The fractures were mostly at the thoracolumbar region (74% between T12 and L2) and regarding the type, 68% were A3 or A4 in the AOspine classification (Fig. 1). The mean local kyphosis (LK) was 5.5° ± 4.4 [0; 22] and the mean regional traumatic angle (RTA) was 2.9° ± 8.5, [−8; 29].

### Post-traumatic disc analysis

**Figure 2:**
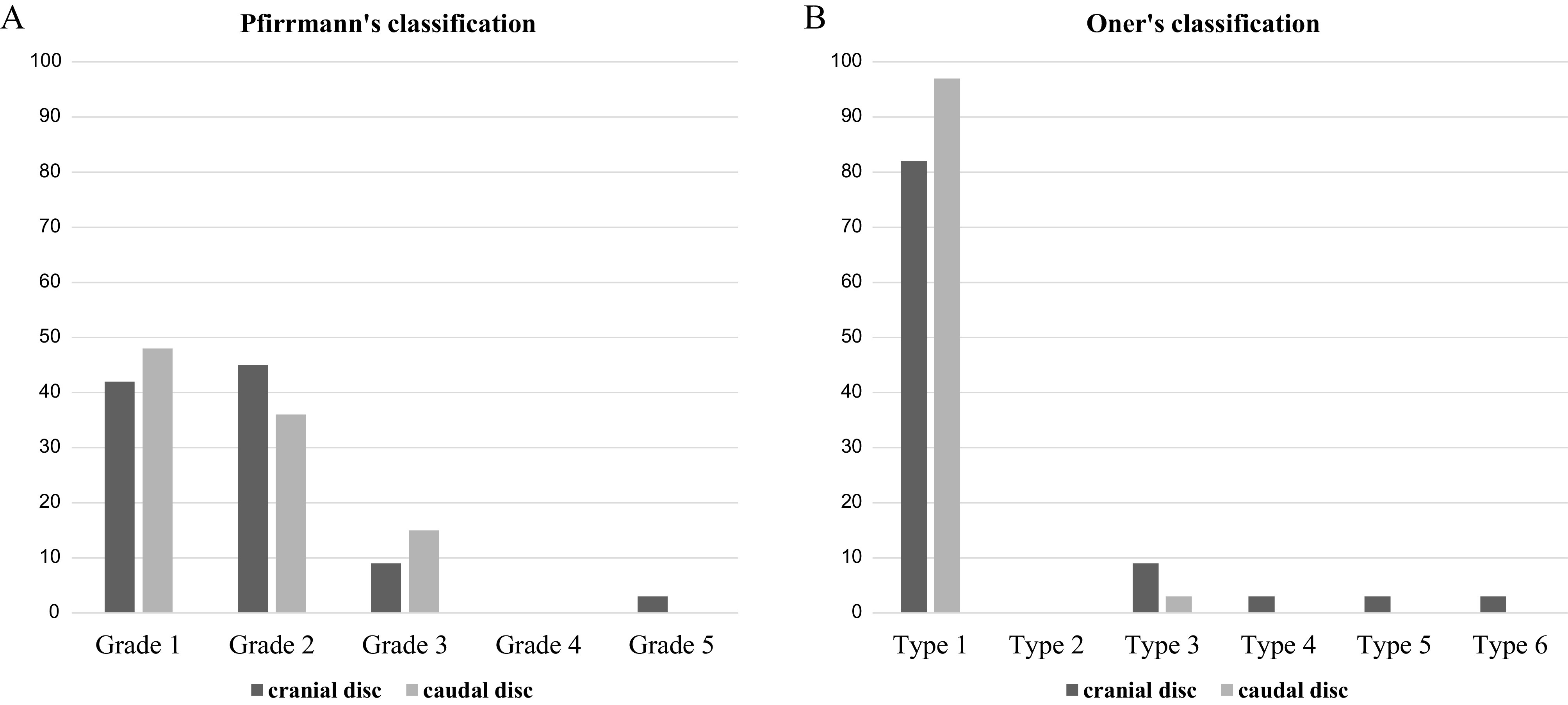
Histogram showing the percentile distribution of disc types according to (A) Pfirrmann’s and (B) Oner’s classification, in immediate post-trauma.

We analyzed 64 discs in T2 MRI images in immediate post-trauma. Among the cranial adjacent discs, according to Pfirrmann’s classification, 43% were grade I, 45% were grade II, 9% were grade III, 3% were grade V, and no grade IV was found, and according to Oner’s classification, 82% were type 1 (normal), no type 2 was found, 9% were type 3, 3% type 4, 3% type 5, and 3% were type 6. Among the caudal adjacent discs, according to Pfirrmann’s classification 48% were grade I, 36% were grade II, 15% were grade III, and no grade IV or V was found, and according to Oner’s classification, 97% were type 1 (normal) and 3% were type 3 (Fig. 2).

In conclusion, according to degenerative aspect, there were 3% (1 patient) of cranial disc and none of the caudal presenting severe degenerative signal at the immediate post-traumatic MRI. Despite this cranial disc degeneration, the patient had a fixation with no fusion.

### Long-term disc analysis

**Figure 3:**
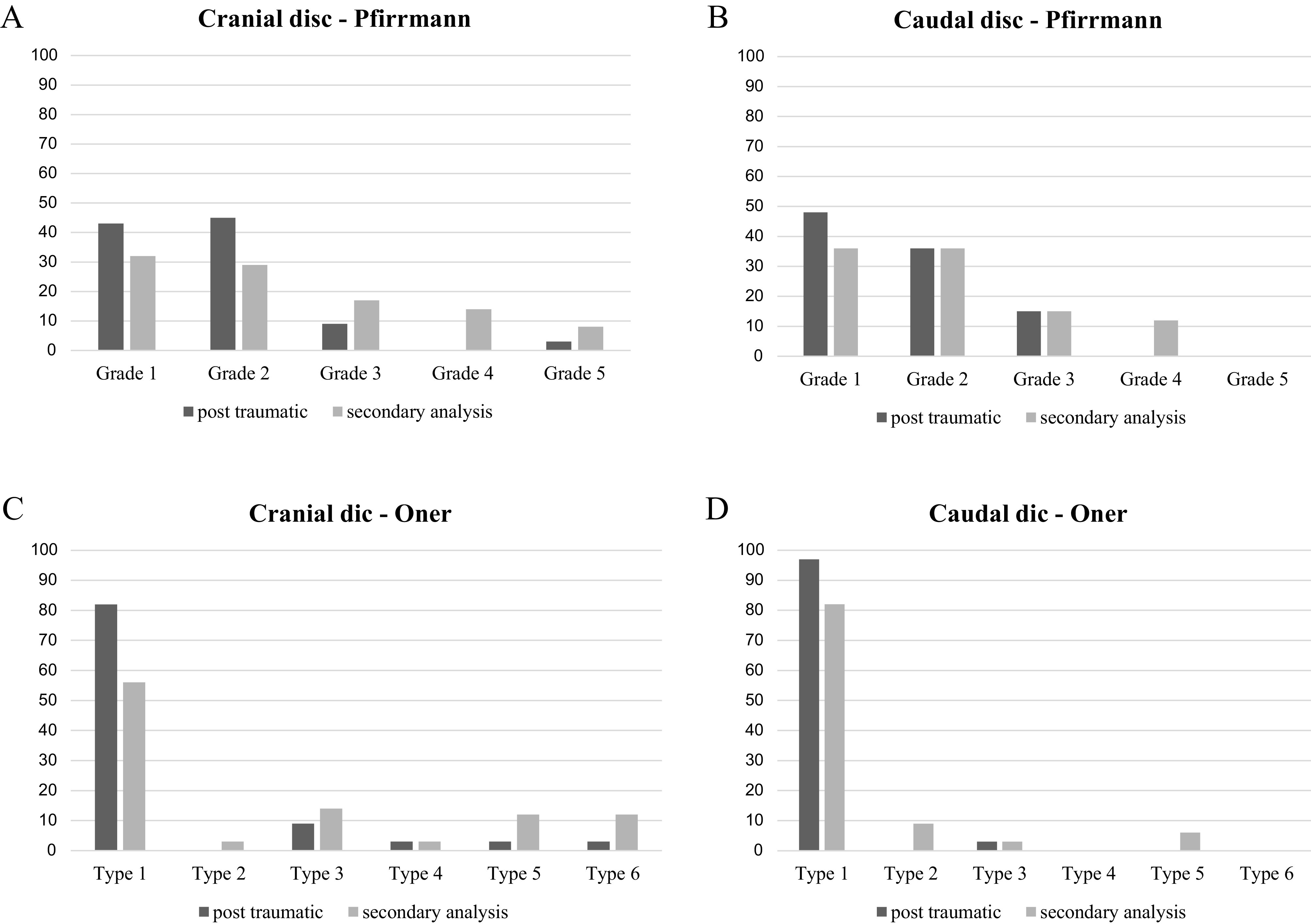
Histograms showing the evolution of the percentile distribution of (A) cranial and (B) caudal adjacent discs types between the immediate (t0) and the secondary analysis (t1) according to (C) Oner’s classification and (D) Pfirrmann’s classification.

**Figure 4:**
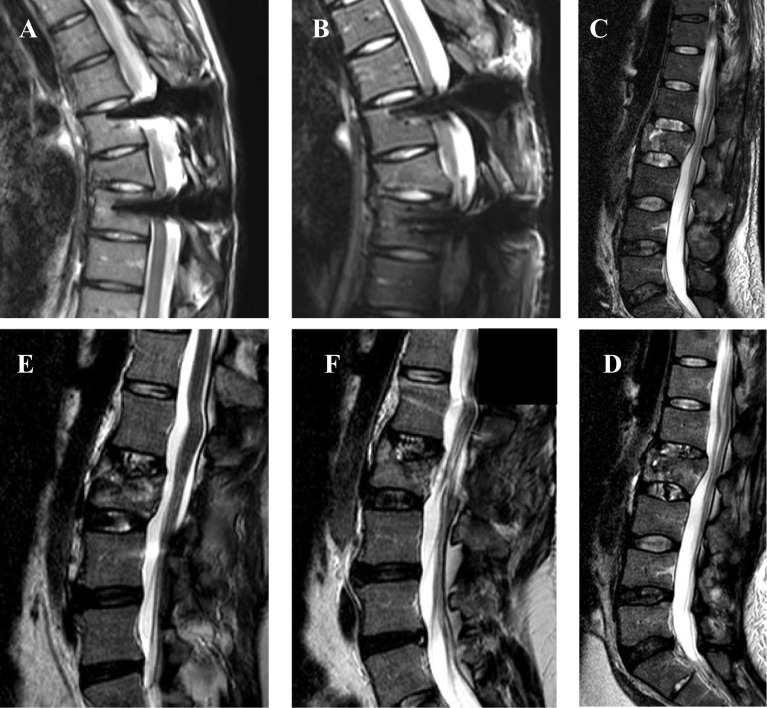
A and B: T2-weighted sagittal MRI images of a T7 fracture (A: post-traumatic and B: at follow-up) showing Oner I and Pfirrmann I adjacent discs. C and D: T2-weighted sagittal MRI images of a L2 fracture showing initial disc lesions (image C), with degeneration on both cranial and caudal discs at follow-up (image D). E and F: T2-weighted sagittal MRI images of a L2 fracture showing a Oner 3 lesion on the cranial disc.

On the second MRI, at a mean follow-up of 32.4 months, among the cranial adjacent discs, according to Pfirrmann’s classification, 32% remained grade I, 29% remained grade II, 17% remained grade III, 14% were grade IV, and 8% were grade V, and according to Oner’s classification, 56% remained type 1, 3% were type 2, 14% were type 3, 3% were type 4, and 12% were types 5 and 6 (Fig. 3: A and C). Among the caudal adjacent discs, according to Pfirrmann’s classification, 36% remained grades I and II, 15% remained grade III, 12% were grade IV, and none were grade V, and according to Oner’s classification 82% remained type 1, 9% were type 2, 3% were type 3, and 6% were type 5 and none degenerated to type 4 or 6 (Fig. 3: B and D). Figure 4 displays examples of sagittal MRI, post-traumatic, and at follow-up.

### Results summary

At follow-up, 22% of the cranial disc and 12% of caudal disc present degenerative signal change on the MRI according to Pfirrmann’s classification, and 15% and 9% according to Oner’s classification.

Statistical analysis showed no association between age, gender, local kyphosis (LK), the regional traumatic angle (RTA), and the modification of Oner’s type in immediate post-trauma or the appearance of a disc injury later after trauma. The only significant factor of modification of Pfirrmann’s grade between post-trauma and last follow-up was the regional traumatic angle (RTA), *p* = 0.034 with Wilcoxon analysis (Table 2).

**Table 2:** *P*-values for clinical and radiological parameters regarding disc lesions in immediate posttrauma (t0) and in the long-term (t1).

a: Wilcoxon analysis; b: Fischer’s exact test			Agea	Gender (M)b	LKa	RTAa
t0	Oner	Cranial	0.65	0.342	0.827	1.000
		Caudal	0.889	1.000	0.748	0.403
	Pfirrmann	Cranial	0.2219	1	0.1083	0.1392
		Caudal	Impossible	Impossible	Impossible	Impossible
t1	Oner	Cranial	0.616	0.054	0.752	0.981
		Caudal	0.195	1.000	0.93	0.07
	Pfirrmann	Cranial	0.5061	0.4082	0.5425	0.3461
		Caudal	0.707	1	0.8644	**0**.034*

* Statistically significant.

There were no differences on changing rates between fractures at the thoracolumbar junction (T12-L2) or below (L3–L5) nor for the cranial or the caudal disc (*p*-values all >0.64 with Fisher’s exact test).

The type of fracture according to AOspine (A1, A2, A3 or A4) had no effect on the disc degeneration (grade I–II–III to grade IV–V of Pfirrmann): the *p*-values after a logistic regression were >0.05 (0.142 for A1, 0.178 for A2 and 0.820 for A3).

### Clinical outcomes

At the last follow-up of 32 months, no patient was revised for disc degeneration. There was no significant loss of sagittal correction between the immediate postoperative and at the last follow-up (+0, 9° ± 1.71 [−2; +5]), (*p* = 0.38).

## Discussion

This study has highlighted the low rate of immediate post-traumatic disc injury after type A thoracolumbar fractures. Only three percent (1 patient) of the cranial discs and none of the caudal discs had severe signal abnormality on the initial MRI. In addition, only 22% (cranial) and 12% (caudal) of these discs will degenerate according to Pfirrmann’s classification with no influence of the AOspine type of fracture or its level. At 32 months of follow-up, no significant correction loss was seen, and no patient was revised for disc degeneration. These results plead in favor of not performing a spine fusion in AOspine A fracture, but rather to achieve reduction of a vertebral fracture.

However, there are some limitations in our study. The number of patients included was limited by the difficulties encountered to have the preoperative MRI, a great part of these patients being polytraumatized and had to be treated in emergency. Another limit of this study, difficult to overcome, is the lack of knowledge of the disc signal before trauma. Obviously, we cannot have at our disposal MRI before trauma and hence we cannot confirm the absence of disc degeneration before trauma in our patients. Several authors have studied the prevalence of disc degeneration among volunteers. According to studies, 6–63% of asymptomatic young adults had disc degeneration [19, 20]. Cheung et al. showed a correlation between age and disc degeneration with a 40% prevalence in volunteer subjects younger than 30 years old up to 90% for subjects over 50 years [21].

Nevertheless, the existing literature on this subject is still contrasted [10, 13–15, 22, 23]. But the methodology and focuses of these studies are variable. For example, Ghanem et al. showed changes in signal intensity in the majority of discs adjacent to traumatic vertebral fractures; however, they analyzed both type A and B fractures and showed that some discs were abnormal on MRI while discography demonstrated no disc injury[22]. Wang et al. analyzed 26 patients with two MRI: immediate post-trauma and after implant removal and found similarly a moderate degeneration of the cranial adjacent disc and no caudal adjacent disc degeneration; but they only analyzed A3 fractures [13]. Our study has the advantage of having a larger number of discs analyzed with two different grading systems (showing similar results), on all type A fractures only and at two times of analysis.

Regarding cranial discs, the morphological change of aspect may be due to the slipping of the disc on a non-reduced endplate, which is also suggested by the influence of RTA on disc degeneration. These kinds of discs that appear with a loss of signal intensity and a loss of disc height on MRI are probably the most pathogenic. This is in agreement with Fürderer et al. who showed that 81% of the discs with initially normal T2 signal showed the same signal after implant removal (after an average of 10 months) [24]. One of the hypotheses to explain disc degeneration observed after a trauma is the calcification of the endplate which leads to a loss of nutrient supply of the avascular intervertebral disc. Loss of oxygen and glucose in the disc and the inability to remove lactic acid lead to cell death and disc degeneration [25]. On the other hand, the greater frequency of injury of the cranial discs compared to the caudal can be explained by the greater fragility of the upper vertebral endplates, combined with the higher intensity of loading charges supported during trauma compared to the inferiors [26]. Hence, the loss of correction after treatment that may have been attributed by many authors to a disc lesion, is for us related to a lack of correction of the upper vertebral endplate and to a secondary intrusion of the disc in the residual depression, which may explicit the morphologic changes observed in some discs (Oner’s types 3, 4 and 5) [15]. Therefore, the objective in the management of type A fractures should be the reduction of the fractured vertebral body to avoid this intrusion. The resection and fusion of the adjacent discs to the fractured vertebra could be avoided. Moreover, because it often involves young patients at the thoracolumbar junction or below, it seems mandatory to maintain the segmental mobility of the spine and therefore to propose implant removal when possible. In fact, several studies showed that the segmental mobility of the lumbar spine can be almost totally restored after implant removal, especially if removal occurs before 12 months [27–29]. That would also prevent from implant failure and adjacent segment disease.

## Conclusions

It seems logical, in type A fractures, to tend to reconstruct the vertebral body, and more specifically the superior vertebral endplate while maintaining the integrity of the over- and under-lying discs. After a CT scan that confirms the fracture healing, an MRI seems useful for assessing the signal of adjacent discs to the fractured vertebra. The analysis in particular of the cranial disc is possible and allows evaluating its possible degeneration. In the case of osteosynthesis, if the disc is healthy, entire implant removal can be performed. In the case of disc degeneration, partial removal of the material associated with segmental fusion at the injured disc level may be proposed. There remains the question of the correlation between disc degeneration diagnosed on the MRI and the presence of back pain. Further researches combining radiological evaluation and long-term clinical scores are warranted to clarify this point.

In any case, long-term follow-up of the patient even after implant removal is crucial and informing him or her of the different evolution patterns is mandatory.

## Disclosure

The authors report no conflict of interest concerning the materials or methods used in this study or the findings specified in this paper.

## Ethical approval

All procedures performed in studies involving human participants were in accordance with the ethical standards of the institutional and/or national research committee and with the 1964 Helsinki declaration and its later amendments or comparable ethical standards. For this type of study formal consent is not required.
